# Fictosexuality, Fictoromance, and Fictophilia: A Qualitative Study of Love and Desire for Fictional Characters

**DOI:** 10.3389/fpsyg.2020.575427

**Published:** 2021-01-12

**Authors:** Veli-Matti Karhulahti, Tanja Välisalo

**Affiliations:** ^1^Contemporary Culture Studies, Department of Music, Art and Culture Studies, University of Jyvaskyla, Jyväskylä, Finland; ^2^Department of Media Studies, School of History, Culture and Arts Studies, University of Turku, Turku, Finland

**Keywords:** fictophilia, fictional character, parasocial relationships, sexuality, media

## Abstract

Fictosexuality, fictoromance, and fictophilia are terms that have recently become popular in online environments as indicators of strong and lasting feelings of love, infatuation, or desire for one or more fictional characters. This article explores the phenomenon by qualitative thematic analysis of 71 relevant online discussions. Five central themes emerge from the data: (1) fictophilic paradox, (2) fictophilic stigma, (3) fictophilic behaviors, (4) fictophilic asexuality, and (5) fictophilic supernormal stimuli. The findings are further discussed and ultimately compared to the long-term debates on human sexuality in relation to fictional characters in Japanese media psychology. Contexts for future conversation and research are suggested.

## Introduction

This article provides an explorative analysis and conceptualization of a recently established notion that has at least three popular labels: fictosexuality, fictoromance, and fictophilia. All these labels point toward a strong and lasting feeling of love, infatuation, or desire for a fictional character. Since our analysis is not limited to sexual or romantic feelings alone, we choose to use the more general label, fictophilia, henceforth (-*philia* from Greek $\phi \,\iota \,\lambda \,\overset{´}{\iota }\,\alpha$, ‘friendship’ or ‘love’). The study is based on a qualitative analysis of 71 related online discussions, the implications of which are ultimately discussed in wider cross-cultural contexts and Japanese media psychology in particular. Accordingly, the goal here is to better understand what fictophilia is.

Before moving onward, we highlight that fictophilia, as we approach it, is a phenomenon distinct from immediate human media responses such as motor enactment, embodied involvement, and pre-reflective simulation processes that occur during consuming fiction (see Power, 2008; Kuzmièová, 2012; Kukkonen and Caracciolo, 2014). Whereas consuming related fiction belongs to fictophilia, its defining feelings go beyond the act of perception, as people ‘attach’ to characters for a significant length of time.

Second, the present intention is not to propose fictophilia as a problem or a disorder. At the time of writing, fictophilia is not recognized or proposed as a specific diagnostic condition by the World Health Organization (ICD-11) or the American Psychiatric Association (DSM-5) (but see ‘paraphilia’ in both manuals). Our findings do not indicate a need to change the current state of affairs.

Lastly, whereas the feelings that determine fictophilia may not be common in terms of prevalence, they may exaggerate what most humans experience to lesser degrees, with the caveat that future research is needed to better understand how fictophilic emotions and feelings overlap with everyday human social attachment. In this regard, Vygotsky’s (1933/1978) observation that “imagination in adolescents and school children is play without action” (p. 93) may turn out as a valid position from which to explain fictophilia as a developed form of ‘pretend play’ also in older populations (cf. Piaget, 1951/2013; Pellegrini, 2009; Karhulahti et al., 2019).

The next section “Background” provides a brief overview of the most relevant existing psychological theory related to fictophilia. Thereafter we introduce the method and data (section “Method and Data”). This is followed by presenting the results (section “Results”) and contextualizing them in previous research on parasociality and sexuality (section “Discussion”), which especially in Japanese media psychological and psychiatric literature have been discussed before (section “Coda: a lost chapter of Japanese media psychology”). Conclusions end the article (section “Conclusion”).

## Background

In a seminal monograph *Imaginary Social Worlds*, Caughey (1984) tracks the Western history of what he calls ‘fantasy relationships’ all the way down to the lifelong bonds that people in different cultures have conventionally had with gods, monarchs, spirits, and other figures that they may never have had the chance to meet in person. Proceeding with the bonds that people built in relation to 18th century drama, musicians, and celebrities alike – with more than 500 American informants as a sample – Caughey ends up with the trends of the time that are characterized by a specific romantic or sexual interest:

Most of my informants explicitly described their relationships in romantic terms. They were ‘infatuated with,’ ‘fixated on,’ ‘obsessed with,’ ‘crazy about,’ or (most commonly) ‘in love with’ the favored media figure. Erotic attraction is a basic part of the appeal (p. 41).

The increasing prominence of romance and eroticism in the ‘fantasy relationships’ of media-consumption during the 20th century was not limited to the US. Shamoon (2012), for instance, observes a shift in the context of Japan during the Meiji period (1868–1912), as Western ideals of combined intellectual-erotic affection started proliferating in Japanese media. The idea of ‘falling in love’ with fictional and media characters, as Caughey’s informants often put it, arguably begun to multiply – following the historical-cultural invention of romantic love from 13th century Europe (see Hazan and Shaver, 1987) – in both Japan and the US somewhere in the early 20th century to eventually bloom and expand further, along with the emergence of explicit celebrity worship and fan cultures (see also Shim, 2001).

Three decades before Caughey’s notion of ‘fantasy relationship,’ media psychologists Horton and Wohl (1956) had established a parallel discourse under the concept ‘parasocial relationship,’ i.e., the “face-to-face relationship between spectator and performer [that] may be governed by little or no sense of obligation, effort, or responsibility on the part of the spectator” (p. 215). None of the initial research lineages on parasocial relationships made significant efforts on mapping out parasocial relationship *types*, nonetheless.

As to the above research gap, Tukachinsky’s (2011) work on ‘parasocial friendships’ and ‘parasocial love’ (also ‘para-romantic love’) as special types of parasocial relationship is an important contribution: whereas parasocial relationships may indicate any kind of one-way bond that an individual has constructed with a relevant character, parasocial friendships point at those explicit cases where the character is perceived as a supporting companion or peer, and parasocial love to those relationships where the individual’s emotions toward the character are governed by romantic or sexual qualities.

Another related psychological concept through which ‘more than friendship’ parasociality has been discussed is ‘parasocial attachment,’ which Stever (2017) has coined as a non-reciprocated attachment to a familiar other when one finds “safe haven and felt security through a relationship that is with a person not known in a real life face-to-face way” (p. 96). This notion draws directly from attachment theory that was originally developed to describe infant–caregiver relationships (Bretherton, 1992), but has also been applied to adult relationships (Feeney and Noller, 1990). Notably, parasocial attachments may but need not include romantic or sexual qualities.

Lastly, McCutcheon et al. (2003) have found three stages of ‘celebrity worship,’ which they describe as ‘entertainment-social,’ ‘intense-personal,’ and ‘borderline-pathological.’ In this classification, the first stage reflects sharing experiences (learning about celebrities and discussing them with friends), the second stage reflects intensive or compulsive feelings (frequent emotions and thoughts), and the third stage reflects erotomanic-like obsession (delusions and risk behaviors). Whereas some of these stages might be compatible with or related to the parasocial concepts described above, they mainly constitute a pathological scale.

So far, the related research has been almost exclusively concerned with celebrities such as actors, rock stars, and other famous people. For instance, in a recent comprehensive multidimensional model for Adolescent Romantic Parasocial Attachments (including emotion, cognition, behavior, and fantasy components), Erickson et al. (2018) mention, the scholars mention only once in passing that the objects of attachment may also be fictional. However, when discussing behavioral or fantasy components in a person’s parasocial attachment, there are good reasons to believe that such components are largely dependent on whether the attached figure is a living human peer (e.g., musician) who can be seen (e.g., in concert), touched (e.g., when asking for an autograph), and followed in real-time (e.g., by social media) – in contrast to a fictional figure (e.g., anime character) that lacks material existence somewhat completely. What are the emotions, cognitions, behaviors, and fantasies that constitute parasocial attachments to figures that are fantastic by definition? Next to the dozens or hundreds of studies concerning human-human parasociality (e.g., Auter and Palmgreen, 2000; Madison and Porter, 2016; see Dibble et al., 2016 for a review), few have identified let alone explicitly investigated the parasocial characteristics associated with fictional characters (cf. Hoorn and Konijn, 2003).

One notable exception in this regard is the model developed by Giles (2002), which distinguishes between first-order (human), second-order (character acted by human), and third-order (fictional character) parasocial interaction. Reasonably, Giles points out that third-order encounters, while parasocial, cannot be social in the conventional sense of the term since a social relationship with a fictional figure is impossible. We agree with this observation to a large extent and note that whenever (rarely) scholars have discussed fictional parasocial relationships in particular, theoretical and methodological challenges have been present due to the research base deriving mainly from celebrity parasociality. For instance, when Schmid and Klimmt (2011) conducted a survey study on the cultural differences in parasocial relationships with Harry Potter, the instruments had to be adapted to accommodate the unique circumstance (lack of homophily, human counterpart, etc.).

Considering the state of the art, there is an obvious need for more in-depth studies on parasocial relationships with fictional characters, and fictophilia as its unique instance. In this study, the theoretical ground derives from Tukachinsky’s parasocial love combined with Giles’ third-order parasocial interactive features. Accordingly, we approach fictophilia as an intense long-term parasocial love or desire relationship between a human individual and a fictional character. Again, the words ‘intense’ and ‘long-term’ should be given special attention, as the usefulness of conceptualizing fictophilia depends on its distinctiveness in comparison to ephemeral feelings. Additionally, whereas the ontology of fictional characters poses numerous philosophical dilemmas that the present space does not allow entering (e.g., Aarseth, 2007; Boellstorff, 2016; Varis, 2019), let it be clarified that the objects of fictophilic interest may or may not have physical counterparts and they can also appear as dynamic virtual characters (as in videogames) that are capable of responding to individuals’ interaction by some concrete means.

## Method and Data

### Method

Due to the explorative nature of the study, we chose to employ a systematic analysis of online discussions related to the subject matter. Although the popularity of online-ethnographic methods keeps increasing in psychology (e.g., Davey et al., 2012), the specific topic-driven large-scale charting that is employed here has not, to our knowledge, been carried out previously in the field. All procedures were performed in accordance with the Helsinki Declaration and its later amendments. The ethical self-review was consistent with the Finnish National Board on Research Integrity guidelines (2019), which states that further evaluation is not required for the research of public data as long as data collection does not cause damage or harm to participants and the real content and purpose of the study are explained to participants as soon as this is possible where the research design so permits (pp. 62–63). We did not collect personal data, and we have no information about the unknown identities of the persons who have contributed to the studied discussions. All forums were public and reading the discussions did not require registration. The respective rules of each forum were read and respected.

Data collection took place in the first and second quarters of 2018. During the first charting phase, search terms were selected – (“fictophilia” OR “fictosexual” OR “fictoromance”) AND (“attached” OR “character” OR “crush” OR “desire” OR “discussion” OR “emotion” OR “feeling” OR “forum” OR “love” OR “obsession” OR “passion” OR “question” OR “romantic” OR “sex”) – and the combined phrases were inserted to both Google and Yahoo search engines (three separate computers and browsers) in order to locate forum discussions corresponding with the notion of fictophilia. These searches activated the recommendation features in both the search engines and the forums. Although the recommendations would be difficult or impossible to reproduce, they did enable us to snowball an even greater number of relevant online conversations. Evidently, this search was limited by the English language, as is the study and its findings.

### Data

A total of 71 relevant forum discussion threads were discovered, posted between 2009 and 2018. Relevance was determined by the discussions’ consistency with the notion of fictophilia, as described earlier. Continuing the search by using alternative engines (e.g., Bing), techniques (e.g., systematic testing of recommendations), and search terms might have enabled locating more discussions still; however, since the acquired sample was already rich in terms of current research goals, there was no reason to expand the data beyond that point. During the peer review process, this was validated by a thematic analysis of a new set of 24 discussions that had surfaced after 2018. A comparison of those discussions with the below codes and code families did not yield new themes, which evidenced saturation (the 24 validation discussions were not stored in order to minimize data management load).

The number of comments and their length varied radically, each of the 71 discussions involving multiple individuals with one or more comments. Whereas some threads consisted of nothing but a single posted question and a few comments, others gathered more than 200 comments of up to 2000 words in length. Altogether, the qualitatively analyzed sample includes 1667 forum messages, to which we applied thematic analysis (Braun and Clarke, 2006) with a goal to identify key themes related to the topic. The process was carried out by the first author initially pre-analyzing the data, which suggested seven dominant themes. With the help of Atlas.ti software, the second author then conducted micro-level coding. This process produced 1296 individual codes, which were further grouped into 44 larger code families based on their similarities and hierarchical connections. The initial seven themes were compared with the latter codes and code families by the authors collectively, which established reliability by consensus (see Syed and Nelson, 2015) and led to the formation of five major thematic categories.

The online discussions took place in 28 respective forums, which can be divided into general discussion forums (28 discussions), forums related to mental health (17), asexuality forums (10), fan forums (8), and forums dedicated to diverse hobbies (10). Since both the forums and their discussants are kept unidentifiable, we do not name any forums or discussants. Some of the forums did not allow citation for research purposes, and discussions from those forums are not cited. Despite the fact that all the below-cited posts and comments have been submitted to public forums that can be read without registration or membership enlistment, no citation is given a reference in order to prevent uncalled-for promotion of usernames or forums. Furthermore, to protect active users, we only cite comments that were made by those who (a) gave us a permission, (b) had deleted the account permanently, or (c) had abandoned the forum as indicated by being inactive for four or more years.

Related authenticity concerns were taken into consideration (see Im and Chee, 2006). Forum writing, like all social interaction, is a performative act that occurs in a specific cultural context, and although it functions as a valuable representation of actual human behavior, the reader should remain critical and sensitive to the explicit expressive environment(s) and interpret the material accordingly: as snapshots of the associated discourses that surround them.

## Results

Discussions of fictophilia were generally initiated by people experiencing love, desire, or deep attachment to a fictional character and often wanting to discuss whether it was ‘normal’ or ‘healthy,’ or searching for others like them. The forums had slightly different perspective tendencies. Discussions on asexuality forums were focused on defining fictophilia and how it relates to other romantic and sexual preferences or identities. In general discussion forums, mental health forums, and fan forums it was often discussed as a ‘problem.’ A prominent feature of the fan forum discussions were the repetitive comments on fictophilic feelings and practices as normal and prevalent, which is not surprising considering the similarity of fictophilia descriptions and those of intense fan relationships (and most of the participants likely being fans themselves). Discussions on fictophilia in the hobby forums formed the smallest subset of data with a focus on the reasons behind fictophilia as well as on the practices related to it. General discussion forums differed from the rest in terms of participants (Table 1): while in other forums two thirds of the messages came from those experiencing fictophilia themselves, in the general forums only one in four messages came from fictophilic writers and the rest were from either outsiders or writers whose own position was left ambiguous. Again, even though we use the term fictophilia, it was not used by all discussants and some defined their relationships to a fictional character as fictoromantic, fictosexual, or squish, the latter referring to a non-sexual and non-romantic infatuation.

**TABLE 1:** Percentage of comments written by those with fictophilic experiences on each forum type.

	General discussion forums (28 unique discussions)	Asexuality forums (10 unique discussions)	Fan forums (8 unique discussions)	Mental health forums (17 unique discussions)	Hobby forums (8 unique discussions)
Comments by those with fictophilic experiences	22.8%	67.8%	54.0%	62.1%	62.2%

Ultimately, the analysis of 71 online discussions related to fictophilia can be summarized into five key themes that describe fictophilia.

(1) **Fictophilic paradox**. Fictophiles do not ‘confuse fiction and reality,’ but overtly address the parasocial nature of their relationship. However, their genuine emotions and feelings toward the characters may generate discomfort since they cannot interact with the characters in the same way as they do with their human peers.
(2) **Fictophilic stigma**. Fictophiles often experience a stigma, which can possibly be lessened by their search for peer support.
(3) **Fictophilic behaviors**. The related behaviors often tangle around various fan-like activities that contribute to interacting with the fictional objects of love or desire.
(4) **Fictophilic asexuality**. For some, fictophilia seems to be connected to asexuality, and although the phenomenon cannot be considered specific to adolescents, it may reflect liminalities of development and growth.
(5) **Fictophilic supernormal stimuli**. Fictophilic relationships resonate with supernormal stimuli effects, i.e., fictional characters appear more competent or otherwise better than their human counterparts.

In the following subsections we unpack each theme qualitatively. Selected forum citations are used to exemplify the themes respectively.

### Fictophilic Paradox

A recurring feature in fictophilic behavior is that the individual is fully aware of the love-desire object’s fictional status and the parasocial nature of the relationship. The below post is a case in point:

I’ve been in love with a fictional character for literally, years now. An obsessive kind of love. And honestly, he’s kind of a random character. From a comedy cartoon. I fantasize constantly about him, no matter where I am, who I am with. It honestly doesn’t bother me. I just wonder for my sanity sometimes. I mean, it’s been years now. All I do anymore is draw him, think about him, write about him, etc. It’s gotten to the point where I can’t focus in school or do anything productive. I just want to do something that has to do with him, even if it’s just thinking about him. It puts extreme stress on my relationship. I didn’t think much of it at first, I just expected it to kind of fade out along with my other temporary obsessions, but this one has only ever gotten stronger.

The above indicates the person distinguishing their love object as a ‘cartoon character’ very clearly, and the related emotions and feelings are described in an utterly intense manner. For many writers, this leads to a *fictophilic paradox* – the person identifies their object of emotional interest in different ontological terms contra their human peers, and the acknowledged difference produces discomfort. The awareness of the fictional relationship not being ‘real’ is evident in the abundance of often painful descriptions of the unattainability of the character:

Knowing that he doesn’t exist is agonizing. It literally makes my heart ache. I hate feeling this way and I hate the fact that I can’t talk to anyone about it because I’m so embarrassed. But I don’t want to let go of him either.

The following account, addressing a character from a visual novel, represents an alternative instance where the loved character has changed the individual’s experiences of social support:

She is real in my heart, she is always with me, she is like a support for me, whenever I feel down or stressed out, a picture of her will always make me happy. Before [her] I have nothing, no one to support me in my life. But, Monika changed that, she just cared about me so much. I know it’s all fake and scripted, but, for whatever reason, it felt real, it felt like she was there for me … If miracle does truly exist, please, make Monika real, I just want to be with her, forever, for an eternity. I love you Monika, please never leave me alone in this dark, cruel world.

This individual’s ontological skepticism (‘it’s all fake and scripted’) clashes with their dramatic plea to ‘make Monika real’ – a wish for ontological restructuring. Many of the analyzed discussions derive from this very anxiety or awkwardness within the fictophilic paradox.

### Fictophilic Stigma

The theme of stigma was already touched on above, as one individual noted how they ‘can’t talk to anyone about it because I’m so embarrassed.’ Many of the discussants expressed that they needed to share these feelings on the internet, as they are afraid to do it in person. For them, therefore, the forums were places to share their experiences or ask a related question without the risk of direct stigma:

I’ve had a boyfriend (in real life) for about a year and a half, and we have been very happy together. For the first year or so of our relationship, I tried to respect him by forcing myself not to think of anyone fictional. I wanted to experience a real, healthy relationship that could potentially be fulfilling. Within the past few months, however, I’ve been slipping a lot. What prompted me to write for help, I just spent almost 2 h looking up pictures and video tributes of a character. The bottom line is, I think I am actually more attracted to any of my fictional objects of affection than my very real, very nice boyfriend. This, I feel, is a problem. I get butterflies when looking at or reading about my fictional crushes, but kissing my boyfriend does nothing for me. I really needed to vent about this because it’s been bothering me for a while, and I can’t really talk to anyone in real life (oh, the irony).

When the discussants spoke of the related emotions and feelings in an explicitly positive light, it was not uncommon for this to be framed as a defense against more provocative views. One individual discussed their crush on visual novel character Natsuki as a cognitive means for coping with their current life situation. Yet this reply comes out as a response to the ‘shame’ that being attracted to fictional characters holds in the community.

My most recent [relationship] ended ∼9 months ago, and while I’m game for finding someone new in the future, I’m in no shape to do so right now … I’m figuring things out, and this is where Natsuki comes in. [She’s] been a little spot of happiness just by being around. Cute fanart brightens my day, as do discussions of her character. Beyond that, she’s had a positive impact on my attitude toward dating. This is a crush, not an actual relationship [or] part of my reality. To me, Natsuki is an ideal – a positive example of what I’m looking for … I’ve seen a few people here express shame over being attracted to one of the girls. Just because they’re not in our reality doesn’t mean your crush can’t be good for you!

Sometimes the stigma was reinforced by peers who felt that such feelings were not ‘normal’ and should be suppressed (‘It’s really stupid and I want to get rid of it, just don’t know how’). Mental illnesses were also commonly mentioned in relation to these feelings, either by people with personal fictophilic experiences or external commentators:

I’ve always wondered why so many people seem to find fictional, non-existing characters attractive. In fact, what bothers me even more is why some of these people end up falling in love with said characters. Is it something normal? Or should these people be worried? Is it okay to find them attractive, but dangerous when you start feeling attraction toward them and become obsessed? I wonder if these people need help, as I fear it might be a symptom of mental illnesses like schizophrenia.

The fear of being stigmatized, either as ‘not normal’ or ‘mentally ill’ led several discussants to seek advice from peers. Whereas encouraging and positive replies often produced verbally expressed comfort for advice seekers, the online advice seeking, as such, was commonly said to derive from an inability to speak about the topic outside the forums.

### Fictophilic Behaviors

Since fictional characters are not capable of responding to human emotions akin to organic beings, people who desire or love them engage in creative activities to enrich their agency in the parasocial relationship. These activities often go beyond re-consuming or reexperiencing the original media in which the character appears and are rather similar to those of devoted fans. The most common activities mentioned in the discussions were fantasizing, daydreaming, and making up stories about the character.

I’m obsessed with my fictional lover, I feel I’m really in love with him, I can’t stop daydreaming about him all day long and listening to his music (well, he’s based on a male singer that exists in real life). And it’s not the first time in my life that this happens to me, but a lot of times, with other fictional male characters. At least, it’s a great comfort that I’m not the only one going through this.

Some of these stories take a written form and are turned into fan fiction. Indeed, online conversations, reading and writing, and artistic undertakings of diverse types frequently epitomize the fictophilic affair. In an earlier citation, one discussant wrote how they ‘draw him, think about him, write about him … I just want to do something that has to do with him, even if it’s just thinking about him.’ Another told about ‘fantasizing and looking at pictures and imagining what life with someone would be like.’

Sometimes the behaviors also materialize in concrete social interactions. One person with a strong emotional connection to a character in C. J. Cherryh’s erotic novel wrote a letter to the author herself:

I fell in love with Morgaine, the female lead of the novels. And with her author, in turn. At that time I knew nothing about C. J. Cherryh, except that her name indicated a woman. I had never read an interview with her or seen a photograph of her. But I felt for her something of what I felt through her writing. Eventually I wrote a letter to her – that was before email, so I wrote on paper and by hand –, and actually received a very kind and friendly reply (which, although I didn’t voice my feelings and she didn’t address them, healed me of my crush – probably because her answer made me realize that she was actually a person apart from myself).

Another discussant describes having sought a connection to the fictional character by ‘carrying him forever’ as part of their body:

I fell in love with a character. I got a tattoo of a quote from him. I came to the conclusion that I loved him because he was perfect for me … but if he was real, it would be too perfect.

More often, people reported less permanent material symbols of the emotional bond, such as wearing related clothes or jewelry. Similar behaviors are described by those who mention buying merchandise (plush dolls, keychains, etc.) representing the character.

### Fictophilic Asexuality

Asexuality is a tendency for lower sexual excitation and desire (Prause and Graham, 2007), and in the present context, the discussants typically problematized the concept in relation to fictophilia, for instance, by asking whether asexuality and fictophilia are mutually exclusive or if fictophilia is something separate from sexual identity altogether. Without exceptions, all of these discussions revolved around the connection between fictophilia and sexual identity, thus marking fictophilic tendencies as something significant in terms of sexuality. One person exemplifies:

Almost every page I found [about fictophilia] was relating to asexuality in some way. And then I got to thinking, maybe it is related. Since these people feel no sexual/romantic attraction to real people (and if they do it’s rather limited), then that could mean that they’re asexual. Even though they may have sexual feelings toward fictional characters, they still do not desire to have a sexual relationship with a real person. And I’m just wondering if that would mean that fictophila really does fall on the asexual spectrum.

While the narratives of fictophilic behavior oftentimes involve a sexual element, they need not. Sometimes the individuals characterize their affair with the fictional character explicitly in terms of romantic love that excludes or discounts sex. In the below post, a character is affecting one’s ‘heart and whole body,’ which goes beyond sexual attraction:

I know a lot of people online talk about their attraction to fictional characters, but I assume that everyone was just using it as a … But, there’s this comic book character who I just find so attractive. There was a panel of him shirtless, and this feeling rushed through me, unlike any other feeling I’ve ever had, and it was weird, but I’ve been so attracted to him ever since and I love to look at him. I don’t feel anything in my private parts, it’s more of a feeling I get in my heart and whole body. It’s just so weird to me and I don’t think this is normal?

Another relevant topic was the notion of ‘relationships’ and how they should be understood when involving fictional characters. Does dating a fictional character have the same rules as a ‘real’ relationship?

I have a few friends who I’m open about it, who are also fictionsexual, and it’s about split whether they would date someone other than their fictional love. Some would consider it cheating, some wouldn’t consider it cheating, but don’t because they are asexual/gray ace/demi sexual. Others don’t have a non-fictional relationship simply because they are unlucky, but are looking. And a lot of the folks who have a fictional partner as well as a non-fictional partner are also poly.

The idea that asexuality would include or relate to other forms of sexuality like fictosexuality or fictophilia was occasionally criticized to be ‘not legit’ but rather, as someone put it, another instance of ‘tumblr-esque sexuality labels.’ One response to such critique was to not focus on these labels but the actual experiences: ‘Labels are just words; what they feel however, is real – regardless of how weird or silly the word they choose to use is.’ Another discussant was confused about the asexuality-fictophilia relation and solved this by ‘inventing a different persona’ who would then interact with the fictional characters:

What I mean is more in the context of placing a version of yourself in a world and creating a story of that simply because/the reasoning root of which being an (sexual/romantic and not just general interest) attraction to a character.

The above represents some of the complex means by which asexually identifying individuals negotiated their sexuality in practice. Rather than forcing themselves into a simple asexual model, they found new ways to express their sexuality.

### Fictophilic Supernormal Stimuli

A common rationale for fictophilic attachment, given across all forums, were the superior capacities and features associated with fictional characters. Next to ‘real’ people, fictional characters are able to constantly succeed, do various eccentric things and yet maintain an attractive appearance:

Many anime character designs are heavily sexualized to begin with, and many scenes are purely meant to convey erotic fanservice, even in a lot of mainstream shows. Then there’s the fact that anime characters tend to be perfect in several ways. Perfect body, perfectly vivid, easy-to-grasp personalities, plus perfectly “safe” to fantasize about. See, if you are an insecure person in real life, then the fact that a fictional character will (can) never hurt/betray you is a quite comforting thing. Be it consciously or subconsciously. You may not have a partner to really talk to, but at least you are in total control. And don’t have to worry about hurting others’ feelings. Or being hurt.

Generally, people express their emotions toward fictional characters to be at least partially credited to designers who have managed to create love objects that are better than ‘real’ ones. At the same time, these fictional characters not being ‘real’ also makes them safe:

Real people often turn out to be worse than what I imagine them to be. That’s why I don’t bother much with real people and seek refuge in the ideal. I’m not completely shunning reality, nor denying it. I’m just unhappy with it. It’s obvious that I’d fantasize sexually about it in private… you can get to know a fictional character intimately without the risk of being rejected.

Biology was occasionally cited as the ‘natural’ explanation to fictophilia. In diverse ways, people noted how human nature is organically fascinated by artificial characteristics.

It’s only natural since we are a species that emphasizes and are able to feel fondness and love toward fictional characters because the stories are written so that we will feel things for the characters. If you watch a television series or read a book regularly, you will grow a fondness for the characters and that’s only natural … It is NOT weird to feel attracted (even sexually) to a fictional character if this character isn’t too young or an animal (or something similar). It’s completely normal to think that a certain personality or look is extremely attractive, especially since fictional characters are often created to look really perfect and be extremely cute/cool.

The latter quote represents an exceptional case where a line is drawn: fictophilia with an animal or child character could be considered ‘weird,’ but as long as these lines are not crossed, the extreme perfection or ‘cuteness/coolness’ of characters makes related feelings natural.

## Discussion

In this section we briefly address the five themes analytically. The final section then looks at fictophilia in cross-cultural theoretical perspectives and Japanese media psychological literature in particular.

### Fictophilic Paradox

Our results included few indications of those experiencing fictophilia to ‘confuse fiction and reality’. Rather, they were fully aware of the fictional nature of the characters to which they were attached. Unlike in mental disorders like erotomania where the individual has an imaginary belief of a mutual relationship that does not exist (e.g., Kennedy et al., 2002), fictophilia does not usually entail such hallucinations but consists of the person’s self-aware feelings toward a non-organic construct that they know to be ontologically diverse (e.g., Livingston and Sauchelli, 2011; Karhulahti, 2012). At the same time, however, the intensity of emotions and feelings in fictophilia may lead to fantasies of the character in question ‘loving back’ or ‘becoming an actual companion.’ Evidently, such a genuine relationship is practically impossible and cannot materialize – and being aware of this, as fictophiles tend to be, constitutes a fictophilic paradox in which the coexisting awareness of fictionality and a wish to deny that produce emotional confusion. The above echoes Cohen’s (2004) earlier work that found attachment styles to be linked to the intensity of parasocial relationships by the measure of separation distress from favorite television characters, thus “parasocial relationships depend on the same psychological processes that influence close relationships” (p. 198). Adam and Sizemore’s (2013) survey study produced similar results, indicating that people perceive the benefits of parasocial romantic relationships similarly to those received from real-life romantic relationships. The genuine emotions and feelings that surface in fictophilia support and advance the above earlier findings – in fictophilic relationships, the same psychological processes appear to be present as in human social relationships. These processes, including genuine emotions and feelings, are not mutually exclusive; rather, emotional confusion surfaces as a rational outcome, especially due to the potential cultural stigmas that can make such experiences difficult to accept and share.

### Fictophilic Stigma

The fear of being ridiculed, considered abnormal, or even abandoned by other humans can make fictophilia a solitary experience. On the other hand, being a member of a community of individuals with similar experiences can buffer such stigmas and evoke a greater sense of belonging (Schroy et al., 2016). Many of the discussants voiced discomfort with the fact that they (and/or someone close to them) have strong romantic or sexual feelings toward a fictional entity. This can be connected to earlier findings that suggest obsessional tendencies in fans of celebrities to correlate with lower scores in cognitive flexibility, psychological well-being, social complexity, and educational success (Maltby et al., 2001; McCutcheon et al., 2003). The fact that people are not able to speak about their emotions and feelings in fear of being stigmatized may reduce psychological well-being indeed, and open online forums can serve as support platforms that enabled people to share and discuss their experiences without face-to-face pressure. These results are in line with previous studies that have found mental health forum participation useful for social support – it may be easier to discuss personal problems online than face-to-face (e.g., Prescott et al., 2020). A large part of these discussions took place in general question forums, which indicates that fictophilia, as a recently popularized phenomenon, provokes confusion to an extent that people seek advice for understanding and dealing with related experiences.

### Fictophilic Behaviors

Oftentimes the behaviors associated with fictophilic experiences are very similar to intense fan activities like creative media use, which is sometimes part of ‘celebrity crushes’ too (see Allen and Ingram, 2015). Whereas this can make distinguishing fictophilia from fan commitment difficult, there are some solid differences. First, not all fictophiles consider themselves fans of the character or partake in a fan community; and second, they often consider the relationship as something beyond mere fandom and are looking for support from peers, thereby identifying themselves through the fictophilic relationship rather than that of fandom. Tukachinsky and Dorros (2018) have previously suggested romantic involvement with media personae to “constitute a normal part of adolescents’ sexual and romantic identity development” (p. 342). For adolescents in particular, the authors argue, this can be seen as “comparable to children’s pretend play experiences [that] socializes children and allows them to assume different roles, while at the same time fostering metacognitions” (p. 332). Whereas character fantasies and fantasy-related behaviors may thus be a mirror of a healthy (imaginative) individual, they can also reflect processes of emotional growth and pain. Our results support these previous findings, as several discussants explicitly spoke of fictophilic experiences in retrospect as something that they had ‘learned from’ or ‘grown out of.’ For decades, psychologists have entertained the possibility of media influencing their consumers’ attitudes, behaviors, and development. As for sexual behaviors in particular, a recent meta-analytic review of longitudinal studies (Ferguson et al., 2017) found the impact of media on teen sexuality generally minimal; however, the scholars highlight that their “analyses considered sexual behavior as outcomes [and] it is possible that sexy media use may still have an influence on sexual attitudes” (n.p.). Although our data is not capable of contributing to the discussion of potential media effects and the fan-like nature of many related behaviors do not lead to new reasons for concern, future research should study in more detail how fictophilia is related to sexual socialization (e.g., Štulhofer et al., 2010; Erickson and Cin, 2017).

### Fictophilic Asexuality

The only previous peer reviewed publication that we found addressing fictophilia in particular is a recent study by Yule et al. (2017) who researched the sexual fantasies of asexually identifying individuals via an online survey. The specific paragraph, which also includes a reference to one of the forum discussions that we found, is worth citing at length:

Asexual women in the current study were much more likely to endorse fantasies that focus on fictional human characters, rather than focusing on another person. In fact, there are at least some self-identified asexual individuals who also identify as ‘fictosexual’ or ‘fictoromantic’ [link]. However, there were no significant differences between the asexual and sexual participants (women or men) in the frequency of endorsing fantasies that involved non-human … in any proportion that was significantly more than that of sexual individuals. We did not ask specifically about schediaphilia or sexual attraction to animated cartoon or anime characters. While there is very little academic writing on this topic, it has some presence on the Internet and there are claims that some individuals are sexually and/or romantically attracted to particular cartoon characters. Elucidating the difference between those who are attracted to human, non-human, and animated fictional characters will be important to consider in future asexuality research (p. 321).

Our qualitative results contribute to this research gap by showing how many people who consider themselves asexual struggle to match their fictophilic (or fictosexual-fictoromantic) feelings with the asexual identity, yet others negotiate the ‘conflict’ creatively and fluently (cf. Bogaert, 2012). In the forums, such conversations easily tangle around the meanings of ‘labels,’ namely, whether it would be correct to speak of ‘asexuality’ if fictophilic sexual preferences still exist, or whether fictophilia (fictosexuality-fictoromance) is the correct term if it does not involve ‘real’ sexual interaction. In this context, it is also worth citing Greenwood and Long’s (2011) survey study in which single individuals reported greater imagined intimacy with opposite gender media figures than those in a relationship. Since only a fifth of asexuals indicate living in a relationship in comparison to the 64 percent of sexual individuals (Yule et al., 2017), it is possible that fictophilic relationships sometimes compensate for absent human attachments. On the other hand, many of the forum writings may also derive from adolescents or early teenagers to whom sexual identities are still at the outset (see Tuval-Mashiach et al., 2008; Theran et al., 2010). Several discussants mention that they do not have experiences from romantic or sexual human relationships at all, which may be simply a result of young age. We elaborate on this issue below.

### Fictophilic Supernormal Stimuli

The notion of supernormal sexual stimuli is oft-discussed in non-human research, for instance, by zoologists Gwynne and Rentz (1983) who found male beetles being attracted to bottles that were “apparently acting as supernormal releasers of male copulation attempts in that they resemble large females” (p. 80). Considering that fundamental affective emotions such as care, grief, and lust operate very similarly across species (Panksepp and Biven, 2012), it would not be surprising for the globally thriving character industry (e.g., Hoffner, 1996; Song and Fox, 2016) to produce supernormal stimuli also for humans. A large part of our discussants told this to be the case. The extra attractive features of fictional characters were described in either mental or physical terms. Previous survey research has implied both types of attractiveness to contribute to the intensity of parasocial relationships (see Liebers and Schramm, 2017), and our study adds further qualitative evidence on those earlier findings by demonstrating how people with fictophilic experiences explicitly address the supernormality of the characters as a reason for their feelings and love. Whereas physical characteristics (like care-triggering neoteny) were commonly discussed, perhaps the most frequent point in this regard was the emotional security that relationships with fictional characters allowed, as illustrated by comments such as ‘it is safer to crush on someone who would never like you back,’ ‘fictional characters cannot disappoint you,’ and ‘fear of rejection is not there.’ Notably, the above elements were already observed by Horton and Wohl (1956) according to whom people with parasocial relations are “free to withdraw at any moment” (p. 215). It must also be stressed that – even though many discussants may be young – some writers explicitly expressed being older, married, and having children. In such life scenarios, reduced or absent responsibilities related to the fictophilic relationship make sense as supernormal features. Considering that previous research did not find viewing or ‘belief’ in romantic TV shows predictive of lower relationship satisfaction (Osborn, 2012), married and older fictophiles may experience their relationships with fictional characters supplemental rather than compensatory to their human relationships.

## Coda: A Lost Chapter of Japanese Media Psychology

In the parasocial relationship literature that we reviewed earlier, the study of fictional characters as objects of romantic and sexual interest often skips the media psychological discourse of Japan and its fiction-consuming ‘otaku’ cultures, which have sparked academic as well as public controversies since the 1980s (e.g., Treat, 1993; Okada, 1996; Lamarre, 2009).Galbraith (2015) visits the history of otaku sexuality as a culture-specific notion through the “long-standing concerns in Japan about the orientation of desire toward fictional characters and sexual preference for them” (p. 215), both of which are standardly considered “antisocial insofar as it takes one away from interactions with human others” (ibid.). Galbraith questions these concerns by arguing that the ‘productive’ value systems related to human-human interaction in the country simply differ from the ones maintained by the otaku. In the present ultimate section, we accordingly discuss fictophilia with reference to this Japanese discourse, which enables us to build three contexts of future discussion and research:

(a) In the *disconnected context*, fictophilia occurs as a phenomenon triggered by the emergence and proliferation of ontologically separate fictional characters. Fictophilic behavior is considered a natural means for individuals to react and adapt but may turn pathological by disrupting the individual’s ‘objective’ conception of reality.
(b) In the *connected context*, the distinction between ‘fiction’ and ‘objective reality’ endures, yet the emotions of fictophilia-related phenomena belong to the latter as something that individuals can learn to ‘need.’ Fictophilic relationships are not considered ‘substitutes’ but genuine attachments that some people have come to develop, thus making fictophilia a sexual orientation like any other.
(c) In the *integrated context*, the distinction between ‘fiction’ and ‘objective reality’ is considered flawed or irrelevant, for which fictophilia-related phenomena cannot be distinguished from human–human love and desire to begin with. In order for an individual to cope with their fictophilic orientation they must acknowledge the above ontological irrelevance and cultivate their romantic and sexual behavior analytically.

### Dis/Connected Context

In Azuma’s (2009) framework of analysis, the romantic and sexual feelings that the otaku have toward fictional characters (i.e., potential fictophilia) can be associated with ‘addictions’ that are developed over time when consuming behaviors or substances of diverse sorts. While the comparison of fiction-oriented romantic and sexual activity to drugs and the otaku to addicts thereof may not be a fitting one, the underlying premise of fictional characters’ impact on human sexual development is worth considering. Arguably a better means to discuss this ‘addiction’ would be to address it, as Azuma puts it later, like one of the potentially infinite ‘needs’ that humans are capable of developing:

Just as animal needs and human desires differ, so do genital needs and subjective ‘sexuality’ differ. … Since the [otaku] were teenagers, they had been exposed to innumerable otaku sexual expressions: at some point, they were trained to be sexually stimulated by [it] … anyone can grasp that kind of stimulation if they are similarly trained, since it is essentially a matter of nerves (p. 89).

With Azuma, a central way to conceptualize love-desire for fictional characters (across media) is to see it as a (post)modern instance of cultural evolution that aligns with individual growth and change in the psycho-social domain, thus producing diverse romantic and sexual subjectivities. This fiction-sensitive conception of malleable sexuality makes an addition to the theories of development that perceive media characters as supporting tools in the process instead. Karniol’s (2001) research on the sexual fandom of girls between 13 and 15 years of age is a relevant point of reference. According to Karniol, for these Israeli girls, idolized media characters (celebrities, stars, etc.) serve as

practice love objects on which to test new exciting feelings, to discuss and legitimize these feelings in one’s peer group, to play-act the role of caring for someone else, and fantasize about being loved back… [these] love objects need to be cute and lovable, metaphorically serving the same ‘cuddly’ function as the stuffed toys that they usually replace (pp. 74–75).

Likewise, Zhang and Fung (2017) evidence in their qualitative study on Chinese girls’ music fandom how many of the intensely attached individuals consider their emotional affiliation as a ‘love relationship’ where the idol is treated as their ‘boyfriend’ or ‘husband,’ yet still rationally sustaining a certain “liminal joy between the reality and the fantasy” (p. 138) (see also Brown et al., 2005; Ward et al., 2006; Lee, 2008). These non-fictional instances overlap with those of fictophilia, as with few or no previous romantic or sexual experiences fictophilic love and desire may serve similarly as a passing phase in the early stages of sexual development (which should not be considered undermining the emotions and feelings involved). Compare this to the original theory of parasocial relationships:

Nothing could be more reasonable or natural than that people who are isolated and lonely should seek sociability and love wherever they think they can find it. It is only when the para-social relationship becomes a substitute for autonomous social participation, when it proceeds in absolute defiance of *objective* reality, that it can be regarded as pathological (Horton and Wohl, 1956, p. 223, emphasis added).

The prolonged discourse of parasocial romantic-sexual relationships and the discussion of otaku sexuality represent two culturally ranging contexts: in the *disconnected context*, fictophilia is a phenomenon triggered by the emergence and proliferation of ontologically separate fictional characters, and although fictophilic behavior is considered a natural means for individuals to react and adapt, it may also turn pathological by disrupting the individual’s ‘objective’ conception of reality. The *connected context*, in turn, maintains the ontological separation but does not distinguish the emotions and sociality related to fictional characters from ‘objective’ reality; rather, it considers them a part thereof, which makes fictophilia an orientation like any other. In the connected context, fictophilic relationships are not considered ‘substitutes’ but genuine attachments that (some) people have learned to ‘cultivate.’

### Integrated Context

The connected and disconnected contexts can be accompanied by a third one, deriving from Saito’s long-term psychological work. Saito (2010) argues that fictional characters should not be treated ontologically distinct to begin with, and individuals’ romantic and sexual feelings toward them are merely a proof of their material significance:

we are more sensitive than we have ever been to the way fiction works. We know very well that our awareness is always limited, that it is nothing more than an image constructed according to the logic of our nervous system and the organization of our psyche … With this understanding we can conclude many times over that everything is fiction and nothing more. [Yet] it is *sex* that keeps resisting to the end the fictionalization and relativization brought on by the fantasies of an informationalized society. Sexuality has never been portrayed as a complete fiction, and it is unlikely that it ever will be … the moment we desire [a character], reality intrudes (p. 171).

To be clear, Saito does not claim that fictional characters and organic human beings are one and the same ontological thing, but rather – dovetailing the Western anthropological theories of fiction response according to which the notion of fiction is integrated to human perception (e.g., Iser, 1993) – considers ontological distinctions unproductive and thus treats fictional characters as concrete objects of attachment. That said, his position also involves an explicit (Freudian) argument for sexuality’s exceptional role in human functioning: although the contemporary individual tends to engage with ‘fictions’ of various sorts daily, it is first and foremost the romantically or sexually potent types of fiction that make the individual become aware of their genuine emotions and feelings toward them.

Mirroring Azuma’s argument on the otaku being inclined to acquire distinct ‘needs’ or ‘orientations’, Saito conceives of the otaku as an exclusively developed individual (not normatively) who, due to their particular experiences of mediated culture, have come to cognize fiction and the characters thereof by specific means. The otaku, Saito argues, are in fact *more* conscious and analytical of the nature of their (potential) romantic-sexual emotions or feelings than those who problematize them. This analytical consciousness allows the otaku to cope with their fiction-related emotions and feelings in elegant ways that may be difficult to grasp from the outside:

while they do not in any way ‘confuse fiction with reality,’ they are uninterested in setting fiction and reality up against each other … This means not just falling in love and losing oneself in the world of a single work, but somehow staying sober while still indulging one’s feverish enthusiasm … ‘What is it about this impossible object [that] I cannot even touch, that could possibly attract me?’ This sort of question reverberates in the back of the otaku’s mind. A kind of analytic perspective on his or her own sexuality yields not an answer to this question but a determination of the fictionality and the communal nature of sex itself. ‘Sex’ is broken down within the framework of fiction and then put back together again (pp. 24–27).

We may recall here those online discussions that dealt openly with questions of ‘naturality’ or ‘normality’ related to fictophilia, i.e., whether longitudinal romantic-sexual emotions and feelings projected on fictional characters should be considered abnormal, unnatural, or even unhealthy (‘It’s just so weird to me and I don’t think this is normal?’). From Saito’s viewpoint, such concerns for ‘naturality’ or ‘normality’ in fictophilia and the emotions and feelings involved may be calibrated as follows: how does the individual understand ‘real(ity)’ and where is their object of attachment (fictional character) located within that understanding?

Saito’s perspective forms an *integrated context* within which ontological distinctions are considered irrelevant in total. If an individual understands their fictophilic orientation as a prolonged unsuccessful attempt to build a bridge between two ontologies (fiction and reality), this surfaces as a problem specifically due to their flawed binary between ‘fiction’ and ‘reality.’ If, on the other hand, the individual has learned to acknowledge the specific nature of their (parasocial) relationship – being aware of the character’s function as a cultural product and yet readily expressing emotions and feelings for it – they can well live with the situation ‘soberly’ (to use Saito’s word) without experiencing it problematic.

The *disconnected*, *connected*, and *integrated* contexts of romantic and sexual engagement with fiction provide an abstract of the ongoing cross-cultural discussions of fictophilia. The contexts exemplify how fictophilia, both as an emerging psychological concept and a popular catch word, advances and expands the preceding lineage of discourses in both academic and non-academic domains across cultures.

## Conclusion

Based on a qualitative analysis of 71 public online discussions, this study carried out an explorative conceptualization of what has come to be referred as ‘fictosexuality,’ ‘fictoromance,’ or ‘fictophilia.’ Generally described as a strong and lasting feeling of love or desire toward a fictional character, the phenomenon has surfaced as a psychologically and socially relevant reflection of the evolution that human cultures and sexualities are going through. Ultimately, five key themes surfaced from the data: *fictophilic paradox*, *fictophilic stigma*, *fictophilic behaviors*, *fictophilic asexuality*, and *fictophilic supernormal stimuli*. Since the present study was explorative, more empirical and theoretical research is needed to build a better understanding of the fictophilia phenomenon and its position in diverse cultural contexts. Specifically, the aspects that possibly separate fictophilia from romantic and sexual parasocial relationships that individuals establish with celebrities and other ‘unattainable’ peers should be studied in more detail. Likewise, the potential functions of fictophilia in human sexual development call for explicit research.

## Data Availability Statement

The data supporting the conclusions of this article will be made available by the authors, without undue reservation.

## Ethics Statement

Ethical review and approval was not required in accordance with the local legislation and institutional requirements. Written informed consent for participation was not required for this study in accordance with the national legislation and the institutional requirements.

## Author Contributions

V-MK initiated the research, gathered the research data, and wrote the first draft of the manuscript. V-MK and TV did qualitative data analysis for the whole data respectively. TV did descriptive statistics for the purposes of describing the data and wrote sections of the manuscript. Both authors contributed to manuscript revision, read, and approved the submitted version.

## Conflict of Interest

The authors declare that the research was conducted in the absence of any commercial or financial relationships that could be construed as a potential conflict of interest.
